# Modulation of rhythmic visual stimulation on left–right attentional asymmetry

**DOI:** 10.3389/fnins.2023.1156890

**Published:** 2023-05-12

**Authors:** Rong Li, Minpeng Xu, Jia You, Xiaoyu Zhou, Jiayuan Meng, Xiaolin Xiao, Tzyy-Ping Jung, Dong Ming

**Affiliations:** ^1^College of Precision Instruments and Optoelectronics Engineering, Tianjin University, Tianjin, China; ^2^Academy of Medical Engineering and Translational Medicine, Tianjin University, Tianjin, China; ^3^Swartz Center for Computational Neuroscience, University of California San Diego, San Diego, CA, United States

**Keywords:** rhythmic visual stimulation (RVS), steady-state visual evoked potentials (SSVEPs), selective attention, attentional suppression, functional cerebral asymmetry (FCA)

## Abstract

The rhythmic visual stimulation (RVS)-induced oscillatory brain responses, namely steady-state visual evoked potentials (SSVEPs), have been widely used as a biomarker in studies of neural processing based on the assumption that they would not affect cognition. However, recent studies have suggested that the generation of SSVEPs might be attributed to neural entrainment and thus could impact brain functions. But their neural and behavioral effects are yet to be explored. No study has reported the SSVEP influence on functional cerebral asymmetry (FCA). We propose a novel lateralized visual discrimination paradigm to test the SSVEP effects on visuospatial selective attention by FCA analyses. Thirty-eight participants covertly shifted their attention to a target triangle appearing in either the lower-left or -right visual field (LVF or RVF), and judged its orientation. Meanwhile, participants were exposed to a series of task-independent RVSs at different frequencies, including 0 (no RVS), 10, 15, and 40-Hz. As a result, it showed that target discrimination accuracy and reaction time (RT) varied significantly across RVS frequency. Furthermore, attentional asymmetries differed for the 40-Hz condition relative to the 10-Hz condition as indexed by enhanced RT bias to the right visual field, and larger Pd EEG component for attentional suppression. Our results demonstrated that RVSs had frequency-specific effects on left–right attentional asymmetries in both behavior and neural activities. These findings provided new insights into the functional role of SSVEP on FCAs.

## 1. Introduction

When the brain is exposed to a series of rhythmic visual stimuli (RVS, or flicker), it produces oscillatory neural responses which share identical or harmonically-related frequencies with RVS, namely steady-state visual evoked potentials (SSVEPs) (Herrmann, 2001; Srinivasan et al., 2006; Moratti et al., 2007). In comparison with broadband electroencephalography (EEG) signals, SSVEP can provide more stable amplitude, frequency, and phase properties of brain activities in a controlled way. Therefore, it has been widely used in neuroscience research and brain-computer interfaces (BCIs) as a frequency-tagged biomarker that would not introduce functional changes. For example, SSVEPs at different frequencies have been quantified to track the temporal dynamics of attentional processes or probe the neural basis of sensory processing (Mueller et al., 2006; Gregori Grgič et al., 2016; Davidson et al., 2020; Stefanac et al., 2021; Kritzman et al., 2022).

However, the frequency-tagging approach has been gradually questioned for the functional effects of RVS-driven neural entrainment on cognitive processes. Neural entrainment is the process whereby intrinsic neural oscillations synchronize with external stimulus rhythms (Thut et al., 2011; Notbohm et al., 2016; Hanslmayr et al., 2019; Beliaeva and Polania, 2020; Cucu et al., 2022). Previous studies have demonstrated that intrinsic neural oscillations are functionally relevant to cognitive processes, which may serve as a fundamental mechanism to support neural processing but not a meaningless byproduct when the brain works (Buzsaki and Draguhn, 2004; Bueno-Junior et al., 2017; Wstmann et al., 2019; Zhang et al., 2019; Riddle et al., 2020; He et al., 2022). Therefore, neural entrainment to rhythmic inputs, such as electrical, magnetic, or sensory stimulation, has been proposed as a promising mechanism to modulate cognitive functions supported by distinct oscillatory patterns. RVS enables the alteration of oscillatory rhythmic activities, thus showing the potential to causally affect neural processing. For example, it was found that 10-Hz visual flicker could entrain endogenous alpha-band neural oscillations, which could predict periodic behavior modulation in visual perception (Spaak et al., 2014) or impaired detection performance in selective visuospatial attention (Gulbinaite et al., 2017). These findings indicated that the generation of SSVEPs might be accompanied by behavioral changes in cognitive processes. In other words, SSVEPs might be more than simple frequency-following responses but have functional roles in cognitive processes. Yet despite there having been many studies of RVS-driven neural entrainment recently, the understanding of SSVEP effects on cognitive processes remains incomplete.

Functional cerebral asymmetry (FCA), a ubiquitous feature of cerebral organization, has been widely documented in cognitive processes, especially for visuospatial attention (Stevens et al., 2012; Chen et al., 2018). Previous studies have found a diversity of left versus right visual processing asymmetries at the neuroanatomical and functional levels. For example, there are asymmetrical EEG activities in neural networks for attentional processing (Kertesz et al., 1986; Jones and Sliva, 2020; Mulligan et al., 2022) and visual field asymmetries of attention (Brederoo et al., 2019) or perception performance (Carrasco et al., 2022). From the perspective of brain development and evolution, FCA’s emergence increases neural capacity and confers superior brain efficiency of neural processing (Corballis, 2009). Furthermore, the degree or direction of FCA shows a close relationship with the performance of visuospatial attention (Wang et al., 2016) and can be affected by many factors, such as normal aging (Hong et al., 2015), training experience (Deng and Rogers, 2002; Rogers, 2006), and experimental stimulation (Shalev et al., 2018; Chiandetti and Vallortigara, 2019). In these views, FCA provides a functional indicator to evaluate changes in cognitive functions.

Here, we aimed to test the functional effects of SSVEP from the perspective of FCAs in visuospatial attention. Previous studies have demonstrated that the time course of FCAs can be reflected by SSVEPs (Martens and Hübner, 2013). By virtue of stable spectrum and high signal-to-noise ratio, the frequency-tagged SSVEP is able to indicate the asymmetrical allocation of visual attention (Zhang et al., 2022). However, to date, the functional effects of SSVEPs on FCAs have been rarely noticed, which limits our understanding of the mechanism underpinning attentional asymmetry. To this end, we devised a novel EEG paradigm in which participants were required to perform a lateralized visual discrimination task while exposed to a series of task-independent RVS backgrounds. FCA analyses were performed on both behavior and EEG responses in target discrimination. Notably, the applied RVS background was evenly distributed in the left and right visual fields (LVF and RVF) and would theoretically induce SSVEP responses in bilateral cerebral hemispheres with scalp EEG recording (Herrmann, 2001; Zhang et al., 2022). Therefore, the extraction of left–right asymmetrical EEG could attenuate the interference of SSVEP on the observation of subtle activities related to lateralized attentional processes, which would provide a novel and concise way to reveal the neural effects of SSVEPs.

## 2. Materials and methods

### 2.1. Participants

Thirty-eight (16 males; 23 ~ 27 years old) healthy right-handed adults were recruited for the experiment, which was approved by the Institutional Review Board at Tianjin University. All had a normal or corrected-to-normal vision, and they gave written informed consent before the experiment.

### 2.2. Experimental paradigms and procedure

**Figure 1:**
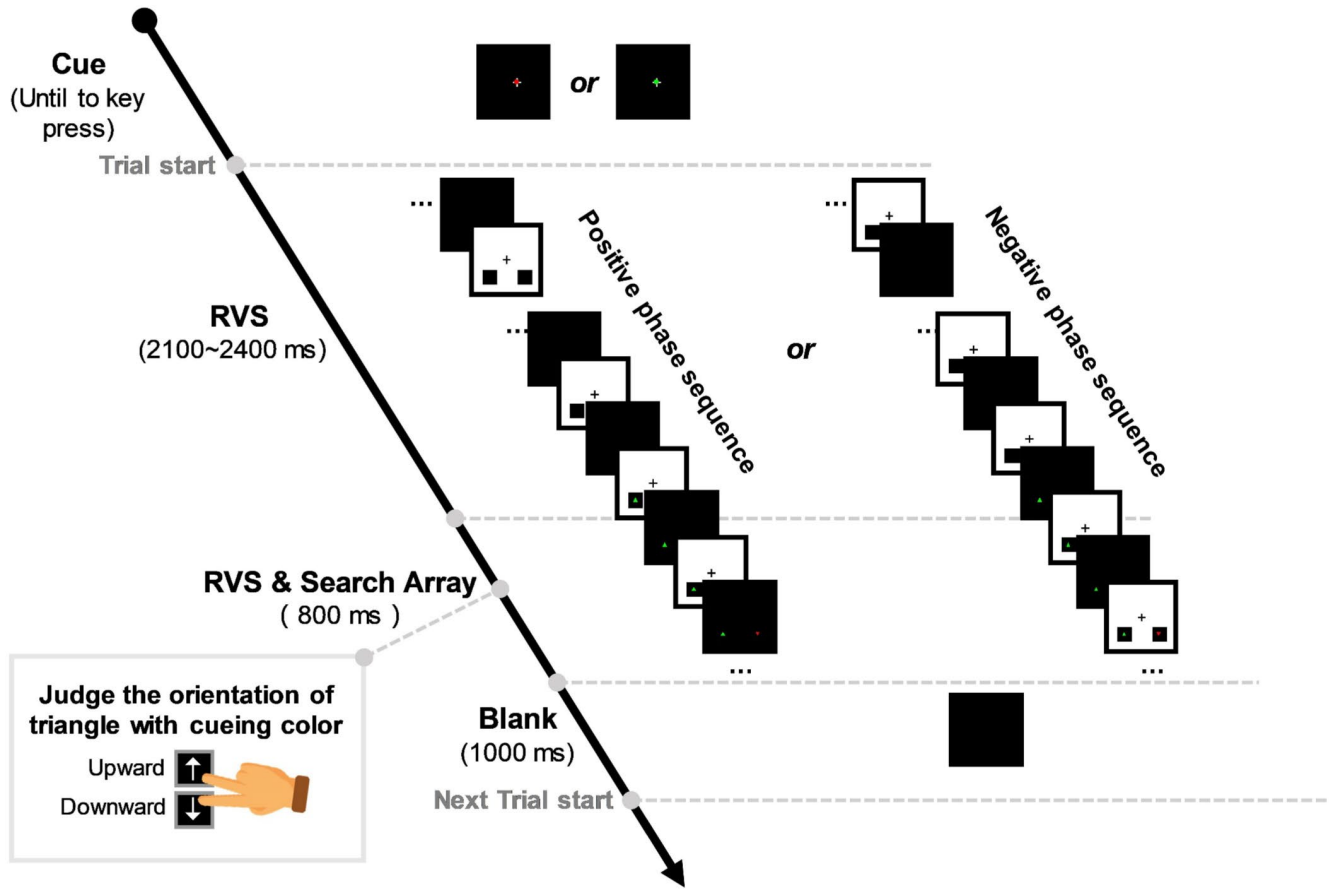
Experimental stimulation sequence. A red/green dot would appear in the centre of the black task background, cueing the target color of each session. Then, a single trial would begin with rhythmic visual stimulation (RVS) flickering in the concentric square area and lasting for 2,900 ~ 3,200 ms randomly. During the last 800 ms of the RVS flickering period, a visual search array consisting of a distractor and a target triangle would appear in the bottom-left and right black squares, respectively. Participants were required to judge the upward/downward orientation of the target triangle with cueing color as accurately and quickly as possible. In each trial, RVS had a frequency selected from 0, 10, 15, and 40 Hz in a random ergodic sequence. To eliminate the effect of RVS phase on target discrimination, we designed positive and negative phases for the 10-Hz, 15-Hz, and 40-Hz RVS sequences. The positive RVS phase indicates that the visual search array appears when the RVS background flashes from black to white, while the negative phase indicates the opposite. There was a 1,000-ms intertrial interval for participants to blink and relax.

Figure 1 shows the time course of the experimental paradigm. In the beginning, a concentric fixation cross would be displayed in the screen centre, with a red (RGB: 255, 0, 0) or green (RGB: 0, 255, 0) dot on it (size: 0.56° × 0.56°), cueing the target color of each session (red or green cue with equal probability). Participants would end the cueing period by pressing the “space” key on the keyboard once they were ready to start the task. Then, each trial would begin with an RVS flickering period lasting for 2,900 ~ 3,200 ms randomly. In this period, RVS was displayed in a concentric square area in the screen (the square edge to the screen centre is 5° apart). Two small black squares (RGB: 0, 0, 0) were embedded in the bottom-left and right RVS flickering area (the square centre to the screen centre is 3.9° apart, dip angle is 45°). During the last 800 ms of the RVS flickering period, a visual search array consisting of a distractor and a target triangle (size: 0.56° × 0.56°) would appear in the centre of the two black squares, respectively. These two triangles were red and green. The target triangle had an upward or downward orientation in the left or right visual field (LVF or RVF), while the distracting triangle had an opposite orientation in the opposite visual field. Participants were required to gaze at the concentric fixation cross and covertly allocate attention to the lateralized target triangle to discriminate its orientation as quickly as possible by pressing the “upward” or “downward” button with the index or middle finger of their dominant hands. In each trial, RVS background would flash between white (RGB: 255, 255, 255) and black (RGB: 0, 0, 0) with a frequency selected from 0, 10, 15, and 40 Hz in a random ergodic sequence. For 0-Hz (the control condition), the RVS square would always remain white (RGB: 255, 255, 255). To eliminate the potential effect of RVS phase on target discrimination, we designed two initial phases, namely positive and negative phases, for the 10-Hz, 15-Hz, and 40-Hz RVS sequences. The positive RVS phase indicates that the visual search array appears when the RVS background flashes from black to white, while the negative phase indicates the opposite. Between trials, there would be a 1,000-ms rest period for participants to blink and relax.

The stimuli paradigm was designed with Psych-toolbox 3.0 package in MATLAB software. Participants sat 75 cm from a 27-inch monitor (spatial resolution: 1,920 × 1,080 pixels, refresh rate: 120 Hz) with a black background (mean luminance: 97.5 cd/m^2^). Each participant completed 24 sessions of the lateralized visual discrimination task in the experiment. There were 28 trials in each session. As a result, we collected 672 trials across 2 visual field × 4 RVS frequency conditions from each participant. All participants’ key-pressing behaviors, such as manual reaction time (RT) and discrimination accuracy, and their EEG signals were recorded for the following within-subject statistical analyses.

### 2.3. Data acquisition and trials selection

**Figure 2:**
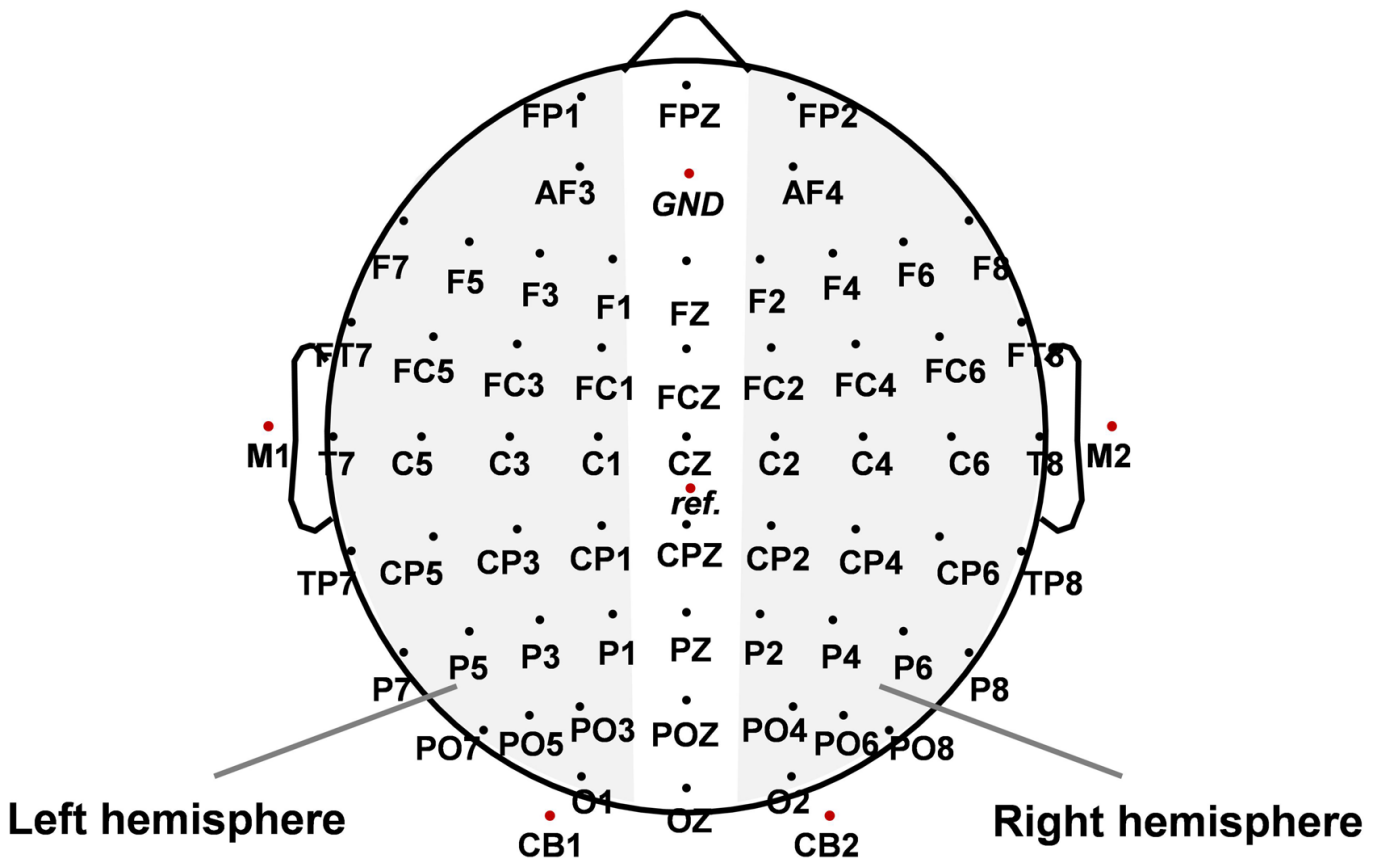
The spatial distribution of scalp electrode sites. *GND* indicates the location of the ground electrode and *ref.* indicates the location of the reference electrode. The paired electrodes in the gray area were used to analyze event-related lateralizations (ERLs).

The EEG data were acquired by a Neuroscan Synamps2 system with a 68-channel cap whose electrodes were placed according to the International 10/20 System (see Figure 2). All recording electrodes were grounded to the middle electrode site between FPZ and FZ. The reference electrode was placed between CZ and CPZ. Eye movements and blinks were monitored by a pair of horizontal electrooculogram (HEOG) channels placed at the outer canthi of both eyes and a pair of vertical electrooculogram (VEOG) channels above and below the left eye. The EEG signal was recorded with a bandpass filter ranging from 0.01 to 100 Hz and a notch filter centred at 50 Hz. The sampling rate of the EEG was set at 1,000 Hz.

The recorded EEG data were offline segmented into trials ranging from −500 ms to 1,500 ms after target-triangle onset. If a trial’s response was wrong or RT was outside the time range of 200–800 ms, it would be excluded from the following analyses. Then, the remains of trials with the top 10% largest HEOG fluctuations would be identified and removed to minimize the impact of eye blinks and eye movements on EEG data. As a result, there were 572.9 ± 5.3 trials available for each participant.

### 2.4. Analyses of behavioral and EEG asymmetries

The behavioral measurements of the proposed task included RT and discrimination accuracy. We further calculated a laterality index of these measurements, allowing us to quantify behavioral asymmetry. The laterality indices for RT ( ${\mathrm{LI}}_{\mathrm{RT}}$ ) and discrimination accuracy ( ${\mathrm{LI}}_{\mathrm{acc}}$ ) were extracted according to the formula (Resnick et al., 1994; Beaton et al., 2007):


(1) $$\mathrm{LI}=\left(L-R\right)/\left(L+R\right)\times 100.$$

where *L* represents the grand-averaged behavioral performance in the LVF condition and R in the RVF condition. The LI values ranged from −1 to 1. Taken $\;{\mathrm{LI}}_{\mathrm{RT}}$ as an example, a positive LI value indicated an RVF advantage of response speed in the lateralized visual discrimination task, and negative LI indicated a LVF advantage. The higher the absolute LI value, the stronger the behavioral asymmetry.

For EEG data, each trial was first filtered by a third-order Butterworth bandpass filter ranging from 1 to 12 Hz. Then baseline correction was performed within the 100 ms time window before target onset. We extracted event-related lateralizations (ERLs) averaged from three symmetrical electrode pairs (P5/P6, PO5/PO6, O1/O2) to analyze hemispheric differences in EEG activities in selective attention. The ERL was calculated as the contralateral-minus-ipsilateral difference of EEG activities recorded in paired electrodes in the left and right hemispheres (LH and RH, see Figure 2) (Oostenveld et al., 2003):


(2) $$\mathrm{ERL}=\left(\begin{array}{l} {\mathrm{EEG}}_{\mathrm{RH},\mathrm{LVF}}+{\mathrm{EEG}}_{\mathrm{LH},\mathrm{RVF}}\\ -{\mathrm{EEG}}_{\mathrm{RH},\mathrm{RVF}}-{\mathrm{EEG}}_{\mathrm{LH},\mathrm{LVF}} \end{array}\right)/2$$

To some degree, the calculation process of ERL waveform suppresses symmetric EEG activity, which is identical to bilateral cerebral hemispheres and visual field conditions. We focused on two ERL components, i.e., N2 posterior contralateral (N2pc) (Luck and Hillyard, 1994; Fuggetta and Duke, 2017) and distractor positivity (Pd) components (Hickey et al., 2009; Suarez-Suarez et al., 2019). Amplitude and latency analyses of N2pc were limited to a time window of 210–270 ms after target onset, while Pd was limited to 300–360 ms. The amplitude of each ERL component was calculated as the mean amplitude within the respective time window. The latency was measured as the time point before which 50% of the total component area was observed in the respective time window.

### 2.5. Statistical analyses

A mixed 2 × 4 analysis of variance (ANOVA) was used to test RT and discrimination accuracy in behavioral performance, with visual field (LVF and RVF) and RVS frequency (control, 10-Hz, 15-Hz, and 40-Hz) as within-subject factors. Then, a one-way ANOVA with RVS frequency (control, 10-Hz, 15-Hz, and 40-Hz) as a within-subject factor was used to test RVS modulation on behavioral asymmetries as measured by ${\mathrm{LI}}_{\mathrm{RT}}$ and ${\mathrm{LI}}_{\mathrm{acc}}$ . The ERL amplitude and latency were separately submitted to a one-way ANOVA with RVS frequency (control, 10-Hz, 15-Hz, and 40-Hz) as a within-subject factor to examine the RVS modulation on EEG asymmetries. The above statistical analyses were subjected to Bonferroni-corrected *post hoc* tests with a significance level of *p* < 0.05.

## 3. Behavioral results

### 3.1. Discrimination accuracy

**Figure 3:**
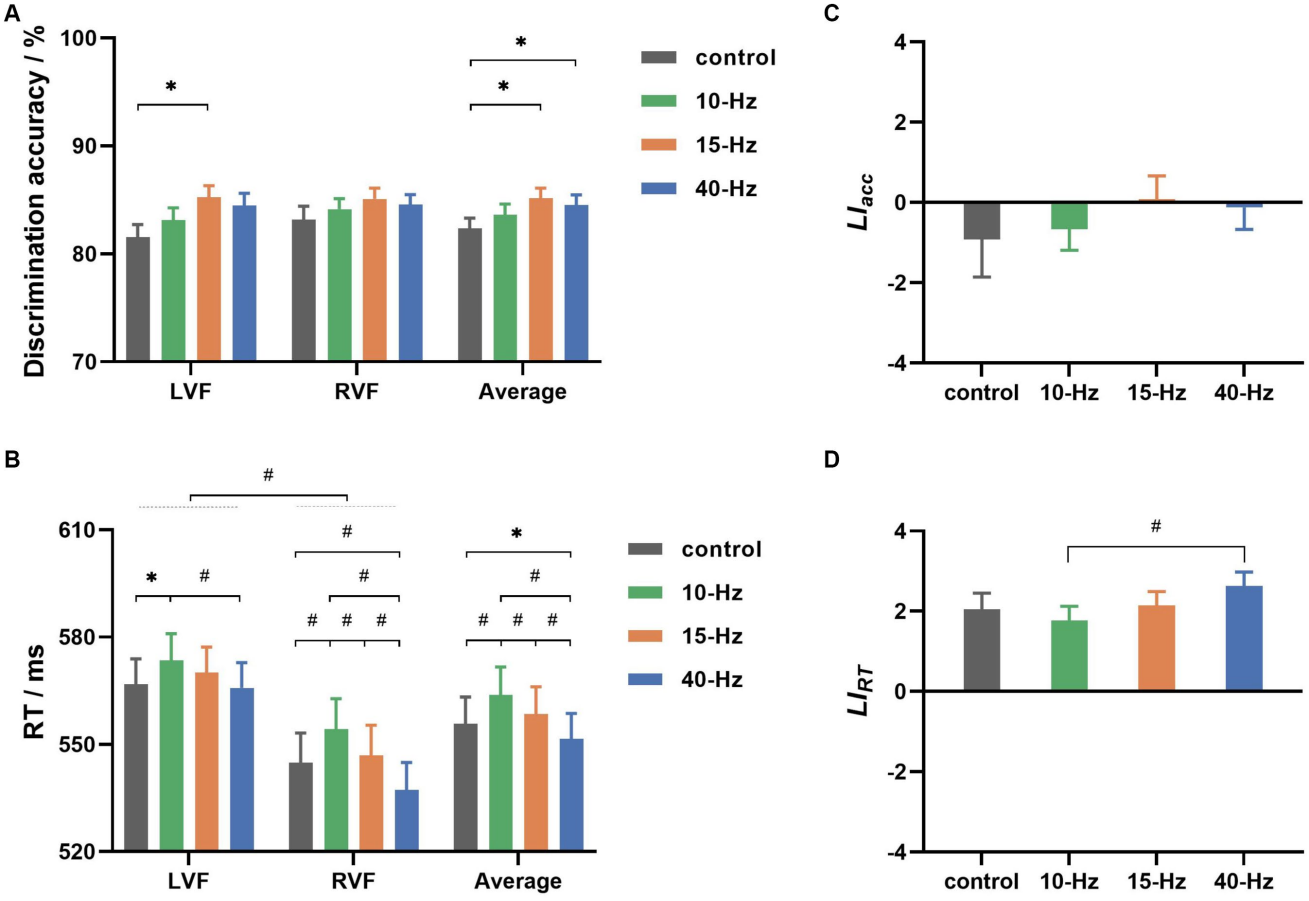
**(A)** The grand-averaged discrimination accuracies for left and right visual fields (LVF and RVF) in 4 RVS frequency conditions (control, 10, 15, and 40 Hz). **(B)** The grand-averaged reaction time (RT) for 2 visual fields in 4 RVS frequency conditions. **(C)** The grand-averaged $LI_{acc}$ values for 4 RVS frequency conditions. **(D)** The grand-averaged $LI_{RT}$ values for 4 RVS frequency conditions. Error bars represent ± standard error; * indicates *p* < 0.05, and # indicates *p* < 0.01 for paired comparisons after Bonferroni correction.

Figure 3A shows that the grand-averaged discrimination accuracy across all participants was 83.92% ± 0.84% (all results are presented as the mean ± standard error). Statistical analysis indicated that participants had similar accuracy between the LVF and RVF conditions (LVF: 83.60% ± 0.91%; RVF: 84.24% ± 0.89%; Visual field: *F* (1, 37) = 1.108, *p* = 0.299, η^2^ = 0.029). However, it varied significantly with RVS frequency (*F* (3, 111) = 5.793, *p* = 0.001, η^2^ = 0.135). Specifically, participants performed better in the 15-Hz and 40-Hz RVS than in the control condition (control: 82.36% ± 0.96% vs. 15-Hz: 85.17% ± 0.91% and 40-Hz: 84.52% ± 0.96%; both *p* < 0.05 after Bonferroni correction). There was no interaction of visual field × RVS frequency (*F* (3, 111) = 0.634, *p* = 0.595, η^2^ = 0.017).

### 3.2. Reaction time (RT)

Figure 3B shows the grand-averaged RTs for all conditions. RVF’s grand-averaged RT was found to be approximately 20 ms faster than LVF’s (LVF: 569.03 ± 7.09 ms, RVF: 545.81 ± 8.18 ms). Statistical analyses indicated that the advantage of response speed for RVF was significant in the lateralized visual discrimination task (Visual field: F (1, 37) = 39.464, *p* < 0.001, η^2^ = 0.516). Furthermore, RVS frequency had a significant effect on the grand-averaged RTs collapsed across visual fields (RVS frequency: *F* (3, 111) = 23.376, *p* < 0.001, η^2^ = 0.387). Specifically, participants responded with a latency of 551.52 ± 7.14 ms for 40-Hz RVS, which was significantly faster than 555.85 ± 7.38 ms for control, 563.84 ± 7.79 ms for 10-Hz RVS, and 558.48 ± 7.59 ms for 15-Hz RVS (all *p* < 0.05 for paired comparisons after Bonferroni correction). Besides, 10-Hz RVS led to significantly slower responses than the control and 15-Hz (both *p* < 0.01 for paired comparisons after Bonferroni correction). Notably, there was a significant interaction of visual field Χ RVS frequency (*F* (3, 111) = 5.437, *p* = 0.002, η^2^ = 0.128). For LVF, only 10-Hz RVS showed a significant modulation effect on RT (10-Hz vs. control and 40-Hz: both *p* < 0.05 for paired comparisons after Bonferroni correction), whereas for RVF, RTs were significantly modulated in both 10-Hz and 40-Hz RVS conditions (10-Hz vs. others, 40-Hz vs. others: all *p* < 0.01 for paired comparisons after Bonferroni correction).

### 3.3. Analyses of behavioral asymmetries

Figures 3C,D show the behavioral asymmetries as evaluated by ${\mathrm{LI}}_{\mathrm{acc}}$ and ${\mathrm{LI}}_{\mathrm{RT}}$ , respectively. As shown in Figure 3C, the grand-averaged ${\mathrm{LI}}_{\mathrm{acc}}$ values were −0.92 ± 0.94, −0.66 ± 0.53, 0.08 ± 0.59 and −0.13 ± 0.54 for control, 10-Hz, 15-Hz, and 40-Hz RVS, respectively. There was no significant difference between them according to statistical analysis (RVS frequency: F (3, 111) = 0.523, *p* = 0.667, η^2^ = 0.014). ${\mathrm{LI}}_{\mathrm{RT}}$ had values of 2.04 ± 0.41, 1.77 ± 0.35, 2.14 ± 0.35, and 2.63 ± 0.37 for control, 10-Hz, 15-Hz, and 40-Hz, respectively, as shown in Figure 3D. These positive ${\mathrm{LI}}_{\mathrm{RT}}$ values indicated that RVF had a group-level advantage in response speed. Statistical analyses revealed a significant RVS modulation effect on ${\mathrm{LI}}_{\mathrm{RT}}$ (RVS frequency: *F* (3, 111) = 5.937, *p* = 0.001, η^2^ = 0.138). Specifically, the grand-averaged ${\mathrm{LI}}_{\mathrm{RT}}$ was significantly higher for 40-Hz than the other conditions before Bonferroni correction but only higher than the 10-Hz condition after Bonferroni correction.

## 4. ERL results

### 4.1. Correlation between ERL components and behaviors

**Figure 4:**
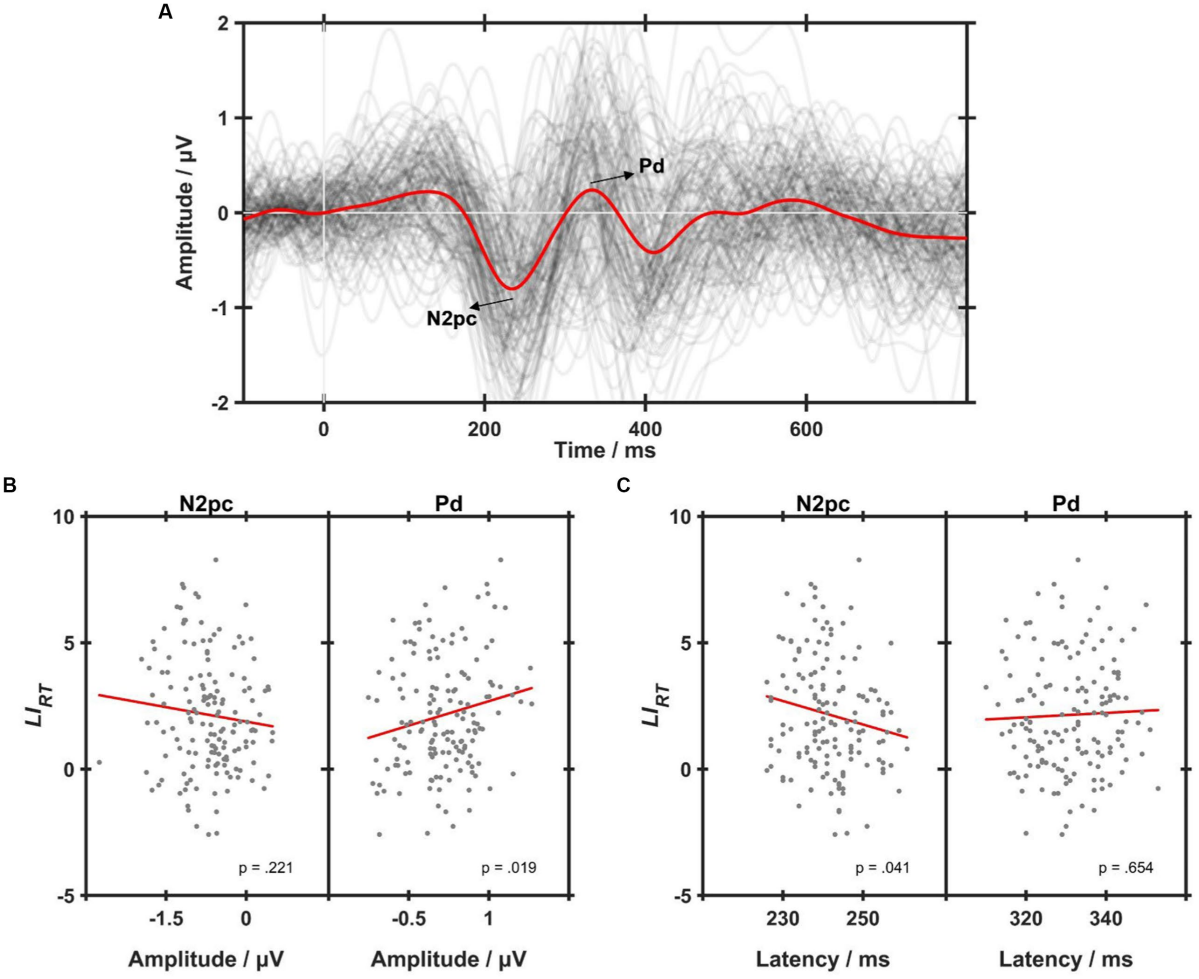
**(A)** The grand-averaged event-related lateralizations (ERLs) across all conditions (the red line). Each dark gray line represents an individual ERL waveform averaged within each condition, i.e., control, 10-Hz, 15-Hz, and 40-Hz. **(B)** Simple lineal regression analyses on the correlations between $LI_{RT}$ values and N2pc (left) and Pd (right) amplitudes. **(C)** Simple lineal regression analyses on the correlations between $LI_{RT}$ values and N2pc (left) and Pd (right) latencies. Each gray dot represents an individual of one RVS condition, and the red line represents the linear correlation fitting curve.

Figure 4A shows the ERL waveforms. Two prominent components, i.e., N2pc and Pd, could be found in the grand-averaged ERL waveform marked red. To explore the relationship between ERL and behavioral asymmetry, regression analyses were performed between the two components and ${\mathrm{LI}}_{\mathrm{RT}}$ , as shown in Figures 4B,C. As a result, the Pd amplitude was found to have a significantly positive correlation with ${\mathrm{LI}}_{\mathrm{RT}}$ (R^2^ = 0.036, *p* < 0.05, see Figure 4B), indicating that a larger Pd corresponded to a larger RT bias toward the RVF. Besides, there was a significantly negative correlation between N2pc latency and ${\mathrm{LI}}_{\mathrm{RT}}$ (*R*^2^ = 0.028, *p* < 0.05, see Figure 4C), implying that an earlier N2pc had a larger $LI_{RT}$ . N2pc amplitude and Pd latency had no significant correlations with ${\mathrm{LI}}_{\mathrm{RT}}$ (both *p* > 0.05).

### 4.2. RVS modulation on ERL components

**Figure 5:**
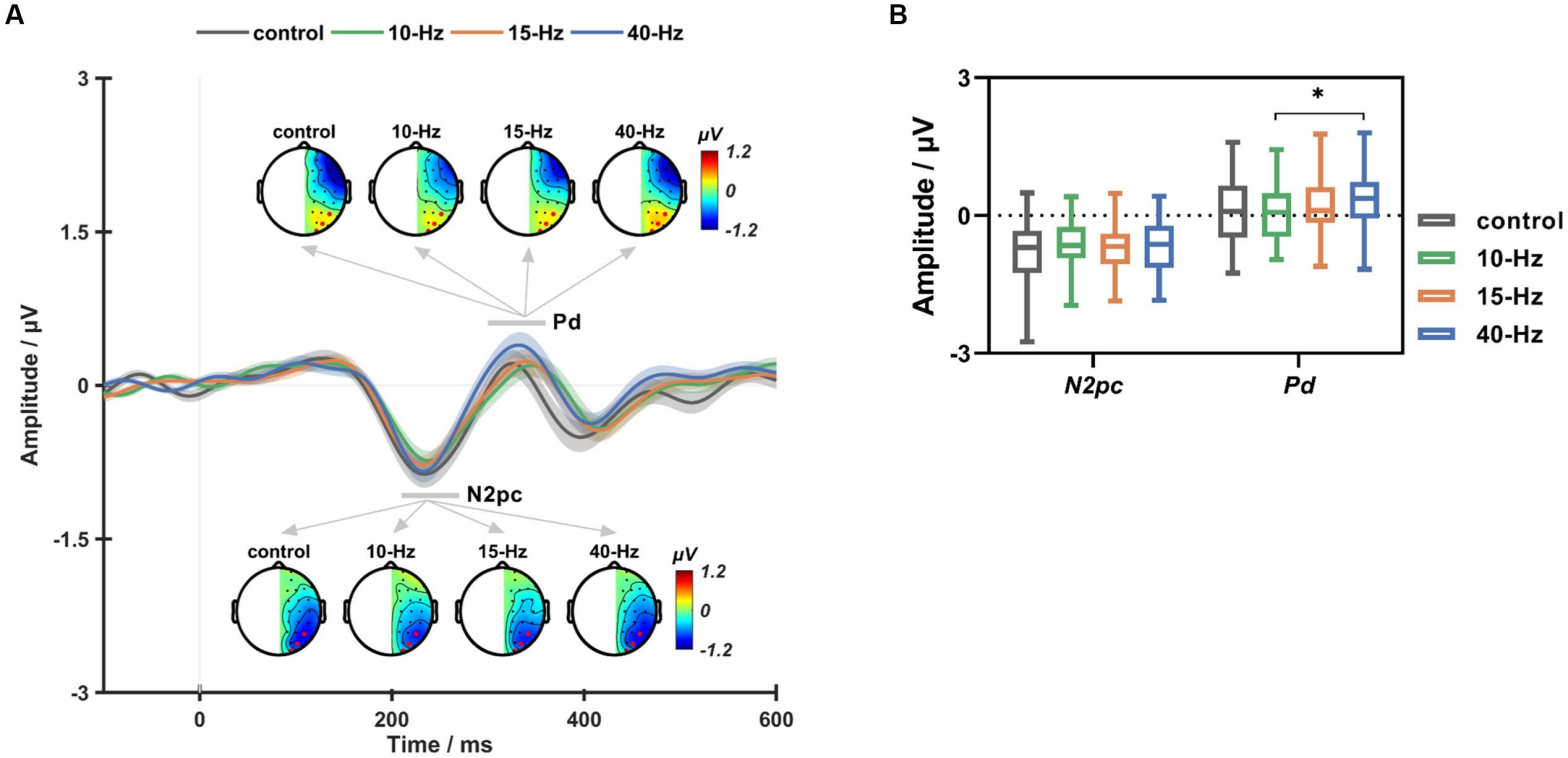
**(A)** The grand-averaged ERL waveforms and topographic maps of N2pc and Pd for the control, 10-Hz, 15-Hz, and 40-Hz conditions, respectively. **(B)** The box plots of ERL amplitude for the control, 10-Hz, 15-Hz, and 40-Hz RVS conditions. Error bars represent ± standard error; * indicates *p* < 0.05 for paired comparisons after Bonferroni correction.

Figure 5A shows the grand-averaged ERL waveforms across all participants and the topographic maps of N2pc and Pd for each RVS condition. As can be seen, the Pd component for 40-Hz appeared to be larger than the others (see Figure 5B). Statistical analyses showed that RVS had a significant main effect on the Pd amplitude (RVS frequency: F (3, 111) = 2.282, *p* = 0.042, η^2^ = 0.071). Moreover, such RVS modulation effects on the Pd amplitude were consistent with that on ${\mathrm{LI}}_{\mathrm{RT}}$ , i.e., 40-Hz had a significantly larger Pd amplitude than 10-Hz after Bonferroni correction (*p* < 0.05). No significant RVS modulation effects were found on the N2pc component.

**Figure 6:**
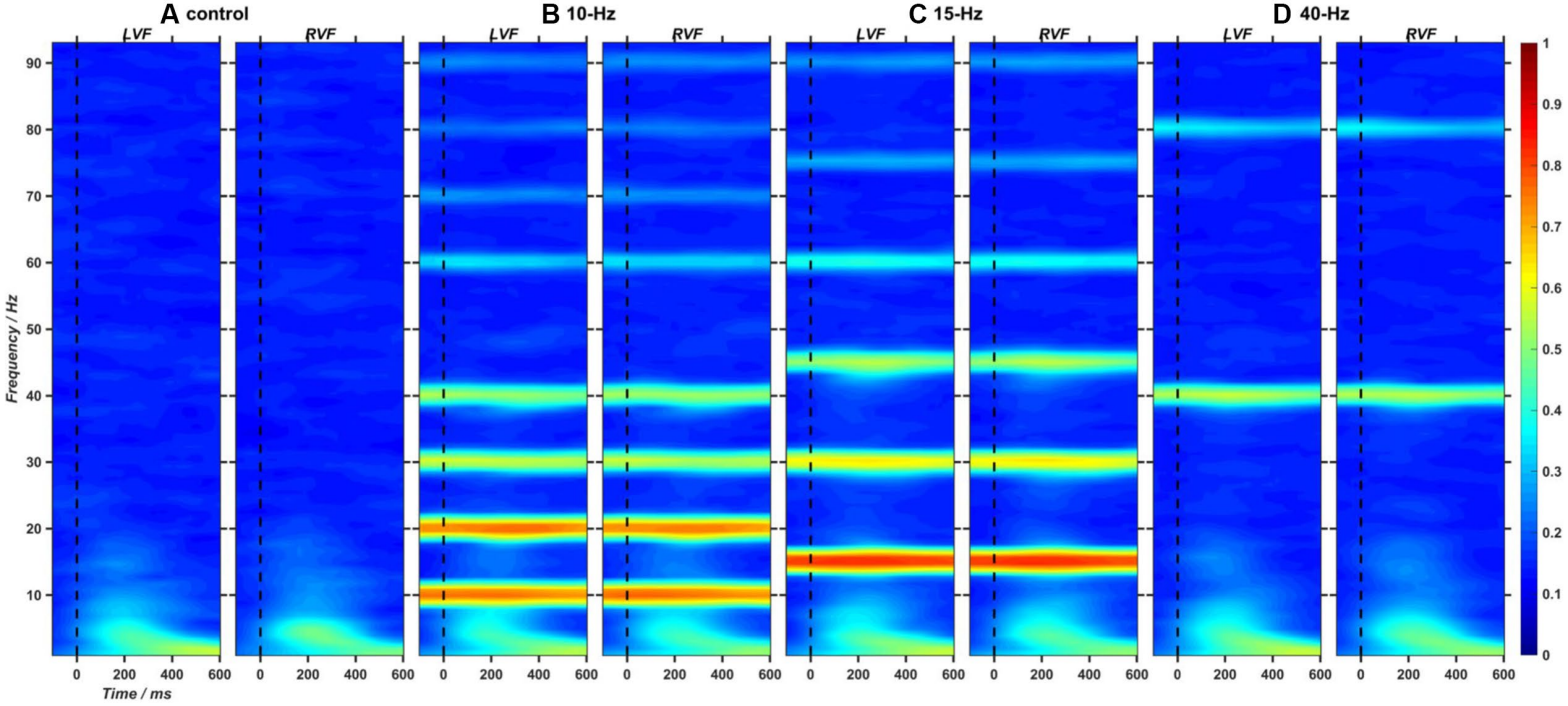
**(A–D)** The 2D images of inter-trial coherence (ITC) extracted from EEG trials at Oz channel and in the left and right visual fields (LVF and RVF) for the control, 10-Hz, 15-Hz, and 40-Hz conditions, respectively.

Using Short-Time Fourier Transform (STFT), we analyzed the target-induced changes in inter-trial coherence (ITC) and event-related spectral perturbation (ERSP) of EEG trials filtered by a third-order Butterworth bandpass filter ranging from 1 to 95 Hz. The ITC measure has been widely estimated as the time-frequency representation of phase-locking strength to the time-locking events of EEG signals (Makeig et al., 2004). To determine whether RVSs could induce frequency-tagged SSVEP response at the corresponding frequency, we presented the two-dimensional (2D) images of ITC measure extracted from the Oz channel in Figure 6. As a result, it was found that 10, 15 and 40-Hz RVS could all exhibit apparent SSVEP responses at the stimulation frequency and its harmonics. Furthermore, consistent with the previous study (Herrmann, 2001), the ITC strength of SSVEP responses showed a decreasing trend with the frequency increased. ERSP measure reflects event-related changes in spectral power (in dB) of EEG data relative to baseline. To investigate RVS modulation on the asymmetrical pattern of EEG power, we extracted the ERL measure of ERSP baseline-corrected with the mean of 100-ms EEG activities before target onset (details in Supplementary material). A one-way ANOVA with RVS frequency as a within-subject factor was performed on the ERL measure of ERSP averaged from three symmetrical electrode pairs (P5/P6, PO5/PO6, O1/O2) and with a time-frequency window of interest (a 150–400 ms window at 1–12 Hz). As a result, no significant RVS modulation effects were found on the left–right asymmetry degree of ERSP measures.

## 5. Discussion

In the current study, we aimed to investigate the functional effects of SSVEP on visuospatial selective attention. To this end, we designed a lateralized visual discrimination task with RVS background and analyzed the left–right attentional asymmetries in behavior and EEG data to evaluate potential functional changes with RVS modulation. Our results indicated that RVSs could shift the fundamental RVF advantage of response speed with frequency-dependent effects. Furthermore, such behavioral changes were in accordance with EEG variations, i.e., the Pd measure of attentional suppression became significantly more prominent for the 40-Hz than the 10-Hz RVS condition, reflecting RVS-elicited enhancement in the suppression of distractor during visual search. These findings indicate that 40-Hz RVS can entrain neural oscillations relevant to attentional suppression and bring behavioral consequences.

### 5.1. Functional cerebral asymmetries in visuospatial selective attention

Both humans and animals exhibit hemispheric asymmetry, which has been documented for many cognitive processes, including visual foraging (Güntürkün et al., 2000), global–local shape processing (Gerlach and Poirel, 2020), and visual-word recognition (Van der Haegen and Brysbaert, 2011). The hemispheric asymmetry contributes a lot to cognitive performance from brain development and evolution (Corballis, 2009). The functional differences between hemispheres in selective attention have been shown to have a lateralized effect on behavioral outcomes (Van der Haegen and Brysbaert, 2011). Moreover, evidence from neuropathological studies indicates that the degree of behavioral asymmetry is closely linked to attentional ability (Poynter et al., 2010). In this study, we found a rightward bias of behavior in the proposed selective attention paradigm. Specifically, the participants had a significant advantage of RVF over LVF in terms of response speed. Our findings align with previous studies on attentional bias, which have shown an RVF advantage in the lateralized recognition task (Vergilino Perez et al., 2012; Bergerbest et al., 2017).

The current task required participants to covertly orient their attention to the target, which appeared in pairs with a distractor. Thus, two distinct attentional processes were theoretically involved in shaping their behaviors, namely attentional selection and distractor suppression (Mazza et al., 2009). Indeed, we observed two relevant ERL components in the posterior brain region, i.e., N2pc and Pd. In light of previous studies, the current N2pc could be explained as a covert deployment of visual attention to the lateralized target during visual search, whereas the presence of Pd indicated an inhibitory process that prevented attention from being oriented to distracting items (Sawaki et al., 2012). The subsequent regression analyses revealed significant correlations between the posterior asymmetry patterns of EEG activities and the behavioral bias, i.e., both the N2pc latency and Pd amplitude were significantly correlated to ${\mathrm{LI}}_{\mathrm{RT}}$ . These findings suggest that the N2pc and Pd components are effective in measuring the attentional bias, which is consistent with previous studies (Salahub and Emrich, 2020; Kappenman et al., 2021).

### 5.2. RVS modulation on left–right attentional asymmetries

An interesting and novel finding in this study was the shift of behavioral and EEG asymmetries in selective attention under different RVS modulations. This study used ${\mathrm{LI}}_{\mathrm{RT}}$ to assess behavioral asymmetry, which showed significant differences among RVS frequencies. Compared to the control condition without RVS, 10-Hz RVS had a slightly lower ${\mathrm{LI}}_{\mathrm{RT}}$ , but 40-Hz RVS led to a higher ${\mathrm{LI}}_{\mathrm{RT}}$ . As a result, the $LI_{RT}$ differences between the 10-Hz and 40-Hz RVS conditions reached a significant level. Specifically, 10-Hz RVS significantly reduced the participant’s response speed for both RVF and LVF, indicating that the alpha-band flicker could impair the task performance in selective attention (Gulbinaite et al., 2017). Whereas 40-Hz RVS significantly sped up the participant’s response only for RVF but not for LVF. The effect of 40-Hz RVS was consistent with previous findings that the gamma-band flicker could facilitate target detection and discrimination (Bauer et al., 2009). Furthermore, it added new insights that 40-Hz RVS could enhance the rightward asymmetry of attention behavior. Consistently, RVS showed frequency-specific modulations on ERL components. Specifically, compared to the 10-Hz condition, 40-Hz RVS induced a significantly larger Pd, indicating increased EEG asymmetry. However, no significant differences were found for N2pc. Considering the functional relevance of these two ERL components, this study indicates that the RVS could influence attentional suppression but not attentional selection. We suggest that RVS might influence behavioral outputs by modulating the suppression process in selective attention.

Our observations, as discussed above, suggest that the applied RVS showed functional modulation on selective attention with behavioral consequences. However, it may be questioned from the following aspects. Firstly, it might be argued that the observed frequency-dependent behavioral effects are not due to the RVS-induced functional modulation but instead reflect the interference of unknown fatigue effects due to prolonged exposure to rhythmic visual input (Dreyer et al., 2017). Indeed, compared with the control condition, the applied RVSs at 10, 15, and 40-Hz can all impact task performance, with a promoting or inhibiting effect on RT or discrimination accuracy. Yet, the applied RVS was evenly distributed in the LVF and RVF. The resulting fatigue effect is expected not to interfere with evaluating behavioral asymmetry. Thus, we suggest that the degree of behavioral asymmetry provides a feasible option for assessing the behavioral effects of RVS modulation. Secondly, because the mental task in this study involved a key-pressing behavior, it is possible that motor-related EEG activity, which overlapped with Pd in the same time window, contributed to the phenomenon of posterior EEG asymmetry. To address this question, we computed ERL on the motor area to investigate the potential influences. Statistical analyses showed no significant differences in the motor-related EEG activity among the four RVS conditions in the Pd time window (details in Supplementary material). Therefore, the observed Pd changes could not be attributed to the contamination of motor-related EEG activity.

### 5.3. SSVEP entrainment from the view of functional cerebral asymmetry

Using time-frequency analyses, this study replicated previous finding that RVS could induce frequency-tagged SSVEP responses at the stimulation frequency of RVS and its harmonics (Herrmann, 2001). In previous studies, SSVEPs have been widely used for studying dynamic neural processes, such as attention control (Wittenhagen and Mattingley, 2019; Kritzman et al., 2022), working memory (Ellis et al., 2006), emotion recognition (Deng et al., 2020), visual information processing (Hansen et al., 2019; Montani et al., 2019) and visual perception (Chicherov and Herzog, 2015). In their views, the frequency-tagged SSVEP can reflect the dynamic neural processes but not influence them, thus making it a good candidate biomarker of brain functioning. However, the frequency-tagging approach is challenged by a contradictory view, i.e., neural entrainment, which argues SSVEP is at least partly generated by the endogenous entrainment of neural oscillations and thus can bring functional changes in cognitive processes (Keitel et al., 2019). It has been demonstrated that rhythmic sensory stimulation (RSS) can entrain endogenous neural oscillations and furtherly affect the subject’s behaviors when they perform some mental tasks, such as attentional selection (Bauer et al., 2009; Gulbinaite et al., 2017), memory (Williams, 2001; Garcia-Argibay et al., 2019; Albouy et al., 2022) and spatial discrimination (Schlieper and Dinse, 2012; Ross et al., 2022). Such behavioral effects caused by RSS could be attributed to the rhythmic shifting of excitability in neuronal ensembles (Lakatos et al., 2008; Calderone et al., 2014) or the interaction between exogenous rhythmic input and endogenous neural rhythm (Spaak et al., 2014; Gulbinaite et al., 2017). Yet, Although there are mounting studies on the rhythmic entrainment phenomenon, the underlying neural mechanism of SSVEP effects remains to be explored.

Using FCA analyses, we demonstrated that the generation of SSVEP were accompanied by functional changes in visuospatial selective attention, which support that SSVEP is more than a biomarker of visual functioning, but also can bring about functional effects via rhythmic entrainment. As one of the most prominent features of cerebral organization, FCA has been widely documented in both human and non-human species (Güntürkün et al., 2000; Hong et al., 2015; Wang et al., 2016; Chen et al., 2018). Abundant converging evidence has revealed the close link between FCA and behavioral output in many cognitive processes, such as visual recognition and cognitive control (Ambrosini and Vallesi, 2016; Schnell et al., 2018). Notably, the degree of FCA is susceptible to many factors, especially environmental stimulation (Shalev et al., 2018; Chiandetti and Vallortigara, 2019). Therefore, we would like to examine whether RVS could impact attention behavior and alter the corresponding neural activities of asymmetry patterns. As a result, we found that RVSs modulated the left–right attentional asymmetries in RT and EEG activities in a consistent manner. These findings demonstrated that RVS could alter attentional processes with asymmetrical behavior and EEG consequences. Furthermore, since the event-related potential is closely related to neural oscillations (Klimesch et al., 2007), the changes in ERL in this study indicate that RVS can entrain functional-relevant neural oscillations. These RVS-induced changes reflect the functional effects of SSVEP, which modulates visuospatial selective attention by altering the left–right asymmetry degree of EEG activities and attention behavior.

In summary, we proposed a novel lateralized visual discrimination task with the background of RVS to explore the functional effects of SSVEP from the view of attention-related asymmetries. As a result, we show that the RVS can influence attentional performance and the degree of left–right attentional asymmetries in behavior and EEG activities. These findings support that SSVEPs play functional roles in neural processing. Furthermore, because FCA has been proposed to be prevalent and relevant to selective attention, we suggest that the observed Pd changes in EEG asymmetries provide new insights into the functional mechanism of SSVEP, which can modulate selective attention by regulating the attentional suppression of distractors during visual search.

## Data availability statement

The raw data supporting the conclusions of this article will be made available by the authors, without undue reservation.

## Ethics statement

The studies involving human participants were reviewed and approved by the Institutional Review Board at Tianjin University. The patients/participants provided their written informed consent to participate in this study.

## Author contributions

MX, RL, and DM designed research. RL and JY performed research. RL, MX, XX, and XZ analyzed data. RL, MX, T-PJ, JM, and DM wrote the paper. All authors contributed to the article and approved the submitted version.

## Funding

This research was supported by the “STI 2030—Major Projects 2022ZD0208900,” the National Natural Science Foundation of China (62122059, 61976152, 81925020, and 62106170, 62106173), and the Introduce Innovative Teams of 2021 “New High School 20 Items” Project (2021GXRC071).

## Conflict of interest

The authors declare that the research was conducted in the absence of any commercial or financial relationships that could be construed as a potential conflict of interest.

## Publisher’s note

All claims expressed in this article are solely those of the authors and do not necessarily represent those of their affiliated organizations, or those of the publisher, the editors and the reviewers. Any product that may be evaluated in this article, or claim that may be made by its manufacturer, is not guaranteed or endorsed by the publisher.

## Glossary

2D: two-dimensional
ANOVA: analysis of variance
BCIs: brain-computer interfaces
EEG: electroencephalography
ERLs: event-related lateralizations
ERSP: event-related spectral perturbation
FCA: functional cerebral asymmetry
HEOG: horizontal electrooculogram
ITC: inter-trial coherence
$LI_{acc}$: laterality index of discrimination accuracy
$LI_{RT}$: laterality index of reaction time
LVF: left visual field
N2pc: N2 posterior contralateral
Pd: distractor positivity
RT: reaction time
RVF: right visual field
RVS: rhythmic visual stimulation
SSVEPs: steady-state visual evoked potentials
STFT: Short-Time Fourier Transform
VEOG: vertical electrooculogram
