# Supplementary material for: Modulation of rhythmic visual stimulation on left–right attentional asymmetry
Source: Front Neurosci. 2023 May 12;17:1156890. doi: 10.3389/fnins.2023.1156890 (PMC10213214; doi:10.3389/fnins.2023.1156890)
Supplement: Supplementary file 1 [file Data_Sheet_1.docx]

Supplementary Material

RVS modulation on the left-right asymmetry of ERSP.

Fig. S1 shows the two-dimensional (2D) images of ERSP extracted from Oz channel and topographic maps of ERSP and ERL measure of ERSP averaged with a time-frequency window of interest (a 150-400 ms window at 1-12 Hz) for the control, 10-Hz, 15-Hz, and 40-Hz conditions, respectively. A one-way ANOVA were performed on the ERL measures of ERSP averaged from three symmetrical electrode pairs (P5/P6, PO5/PO6, O1/O2) with RVS frequency as a with-in subject factor. As a result, it was found that there were no significant main effect of RVS frequency on ERL measures of ERSP, indicating no RVS modulations on the left-right asymmetry of EEG power (F (3, 111) = .103, p = .958, η^2^ = .003).


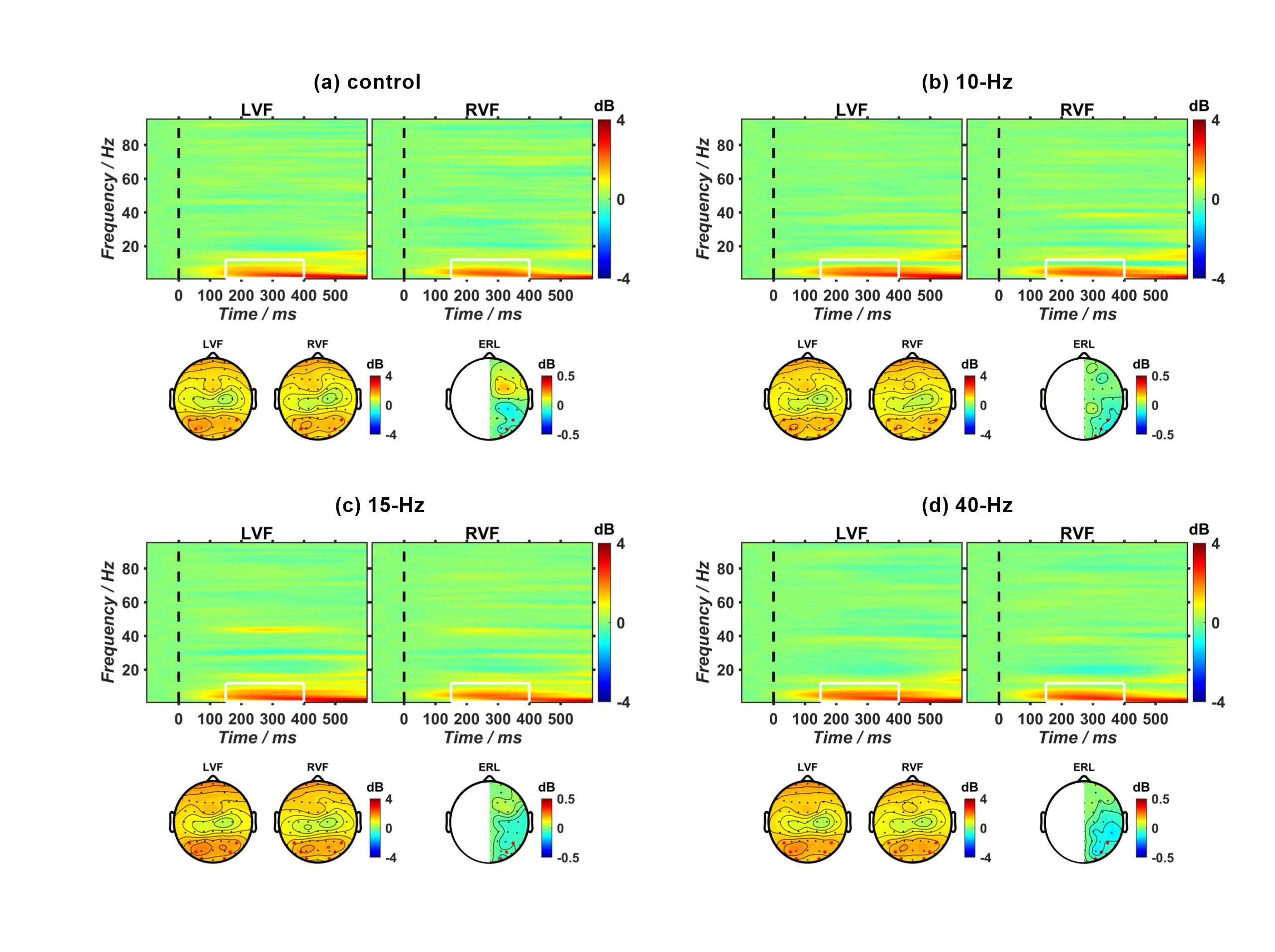


**Fig. S1.** (**a-d**) The 2D images of ERSP extracted from EEG trials at Oz channel and in the left and right visual fields (LVF and RVF) for the control, 10-Hz, 15-Hz, and 40-Hz conditions, respectively. The corresponding topographic plots of ERSP and ERL measure of ERSP averaged with a time-frequency window of interest in 2D-ERSP images (a 150-400 ms window at 1-12 Hz, labeled with white boxes).

RVS modulation on motor-related ERL components.

Fig. S2 shows the motor-related ERL waveforms extracted from C3/C4 electrode pair. In both the N2pc and Pd periods, similar ERL components were found for different RVS conditions. Statistical analyses revealed that there was no significant RVS modulation on both ERL amplitudes (N2pc period: F (3, 111) = .771, p = .512, η2= .020; Pd period: F (3, 111) = .652, p = .584, η2= .017) and latencies (N2pc period: F (3, 111) = .392, p = .759, η2= .010; Pd period: F (3, 111) = .326, p = .806, η2= .009) in the motor cortex.


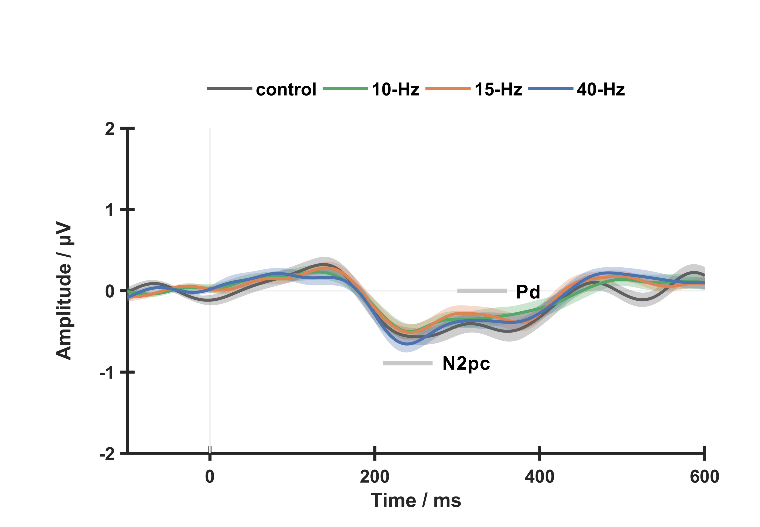


**Fig. S2.** The motor-related ERL waveforms for the control, 10-Hz, 15-Hz, and 40-Hz conditions, respectively. The two gray areas indicate the N2pc and Pd periods, respectively.
